# Banishing the Control Homunculi in Studies of Action Control and Behavior Change

**DOI:** 10.1177/1745691614526414

**Published:** 2014-09-17

**Authors:** Frederick Verbruggen, Ian P. L. McLaren, Christopher D. Chambers

**Affiliations:** 1 School of Psychology, Exeter University, Exeter, United Kingdom; 2 School of Psychology, Cardiff University, Cardiff, United Kingdom

**Keywords:** executive control, action control, behavior change, learning

## Abstract

For centuries, human self-control has fascinated scientists and nonscientists alike. Current theories often attribute it to an executive control system. But even though executive control receives a great deal of attention across disciplines, most aspects of it are still poorly understood. Many theories rely on an ill-defined set of “homunculi” doing jobs like “response inhibition” or “updating” without explaining how they do so. Furthermore, it is not always appreciated that control takes place across different timescales. These two issues hamper major advances. Here we focus on the mechanistic basis for the executive control of actions. We propose that at the most basic level, action control depends on three cognitive processes: signal detection, action selection, and action execution. These processes are modulated via error-correction or outcome-evaluation mechanisms, preparation, and task rules maintained in working and long-term memory. We also consider how executive control of actions becomes automatized with practice and how people develop a control network. Finally, we discuss how the application of this unified framework in clinical domains can increase our understanding of control deficits and provide a theoretical basis for the development of novel behavioral change interventions.

How the brain adjusts behavior in ever-changing environments is an enduring mystery. Scientists have attributed adaptive and goal-directed behavior to *executive control*. This umbrella term is used for the functions of the cognitive system that allow people to regulate their behavior according to higher order goals or plans. This involves organizing, monitoring, and altering the settings of lower level cognitive processes such as stimulus detection and motor programming (Logan & Gordon, 2001; E. K. Miller & Cohen, 2001; Monsell & Driver, 2000; Norman & Shallice, 1986). These functions are critical in everyday life, as they allow people, for example, to resist temptations, overcome habits, or replace actions when required (e.g., when one is driving a car and a pedestrian unexpectedly crosses the street). More generally, executive control has been linked to physical and mental health, school and job success, substance dependence, personal finances, and many aspects of social behavior (Diamond, 2013; Moffitt et al., 2011). Impairments in executive control may underlie many psychopathological disorders, including attention deficit/hyperactivity disorder (ADHD), substance abuse disorders, eating disorders, obsessive–compulsive behavior disorders, and gambling disorders (Bechara, Noel, & Crone, 2006; Crews & Boettiger, 2009; de Wit, 2009; Garavan & Stout, 2005; Nigg, 2001; Noël, Brevers, & Bechara, 2013). The outcome of behavioral change interventions has also been linked to executive control (e.g., Nederkoorn, Jansen, Mulkens, & Jansen, 2007). Thus, it is no surprise that executive control is a central component of many neurobiological models of addictions and of impulsive and compulsive behaviors (Chamberlain & Sahakian, 2007; Crews & Boettiger, 2009; Dalley, Everitt, & Robbins, 2011; Goldstein & Volkow, 2011; Robbins, Gillan, Smith, de Wit, & Ersche, 2012).

In this article, we critically assess the current state of the executive control literature and highlight some pressing issues. We propose a unified framework of executive control and describe how this framework can contribute to our understanding of behavioral change and to the development of new behavioral change interventions that target eating behavior, addiction, and self-control problems more generally. We focus on executive control of actions but also consider how this work could translate to the control of thought and emotion.

## An Army of Control Homunculi

Early research on executive control focused mostly on behavioral deficits after frontal-lobe lesions (for short reviews, see Miyake et al., 2000; Monsell & Driver, 2000). The common finding is that frontal-lobe patients experience problems with organizing and regulating actions; for example, they can become impulsive and are often unable to respond appropriately to changes in the environment. Based on such findings, it was proposed that a critical function of the frontal cortex is executive control of action and thought. After the cognitive revolution against the behaviorists in the 1950s, the concept of an *executive controller* also became very prominent in the cognitive literature. However, in early models of cognition, control was essentially attributed to a unitary “homunculus” who pulls the levers to regulate lower level systems when needed (Baddeley, 1996). Around the turn of the 21st century, many psychologists agreed that this situation was no longer tenable, because homunculus theories may explain what is controlled but not how control is exercised.

The preferred strategy to tackle the “how” question became fractionating the executive controller and determining how distinct control functions regulate behavior. Monsell and Driver (2000) proposed the slogan “Dissolve, deconstruct, or fractionate, the executive! Let a hundred idiots flourish!” (p. 7). They argued that to know how control is exercised, we should identify the very basic processes (the “army of idiots”) that underlie control. In the last decade, great efforts have been made to deconstruct the executive controller. For example, correlational work suggests that there is both unity and diversity in executive control, with at least three distinct executive functions: switching between tasks or mental sets (“shifting”), changing and monitoring representations stored in working memory (“updating”), and suppressing irrelevant information and canceling inappropriate actions (“inhibition”) (Miyake et al., 2000). Many studies have focused on the cognitive and neural substrates of these functions and how they interact with each other. Unfortunately, we believe that this work has not yet succeeded in banishing homunculus theories.

Too often, researchers label cognitive functions as “executive” without questioning the mechanistic nature of the underlying processes. For example, in clinical, social, and cognitive psychology, individual or group differences in controlling actions are typically attributed to variation in the effectiveness of a single control function (e.g., inhibition). Similarly, in cognitive neuroscience, prefrontal brain activation, when people replace one response with another, is often assumed to reflect a form of executive control. However, the community seems to have fallen into the trap of confusing tasks with mechanisms. Many processes contribute to successfully replacing an action. By referring to general constructs such as “inhibition” (or, even worse, “executive control” or “self-control”), we do not explain performance in complex environments—we merely redescribe it. Thus, although many researchers no longer appeal to a single control homunculus, control is often attributed to an ill-defined set of specialized “black-box” homunculi that are assumed to do jobs like “response inhibition” or “updating” without explaining how they do so. We believe that this theoretical strategy of focusing on general functions rather than the underlying processes is limiting progress on the control problem, because in most cases, there are no clear explanations for how the specific functions are achieved.

Furthermore, many (if not most) studies focus on action control in response to changes in the environment. However, various processes that take place on different timescales may contribute to individual and situational differences in the efficacy of control. Preparation or preactivation of subordinate systems that are required to detect a specific stimulus (e.g., a red light), to select a specific response (e.g., hit the brake pedal), or to execute specific action (e.g., move the leg) could have a major influence; similarly, the ability to implement and maintain new rules may prove critical. Finally, action control may evolve over time. The dichotomous distinction between “executive” and “automatic” processes is still omnipresent in the action control literature. Automatic processes are considered to be fast, associative, emotional, effortless, and easily triggered by information in the environment, regardless of the current task goals. By contrast, executive processes are considered to be slower, effortful, rational, and goal directed. But these may be the extremes on a continuum, and control processes that start off as deliberate and effortful can become progressively more “automatic” through experience. By not properly acknowledging the contribution of processes such as preparation and learning, we generate an inherently limited perspective on the cognitive mechanisms behind action control.

**Fig. 1.**
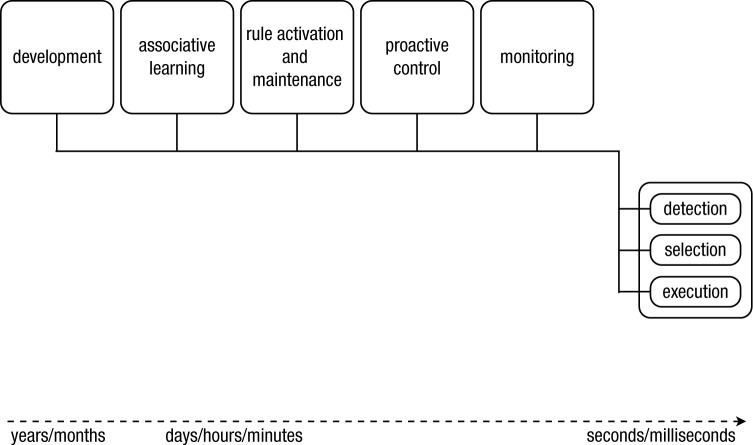
A schematic overview of our framework, which is inspired by Newell’s *Unified Theories of Cognition* (Newell, 1990). We define various forms of behavioral control as an interplay between three basic and computationally well-defined processes (signal detection, action selection, and action execution), which are regulated and influenced by (sets of) processes that take place on different timescales: outcome monitoring, advance preparation, rule acquisition and maintenance, associative learning, and development. We propose that the parameters of all three basic processes (detection, selection, execution) can be influenced by these other processes. In the main text, we discuss each “box” in more detail so as to avoid the introduction of new homunculi.

Our proposed solution for these interlinked issues is a comprehensive theoretical framework of action control and adaptive behavior that integrates research from different areas (see Fig. 1 for a schematic representation). We will focus not only on the functions of the cognitive control system but also on the underlying cognitive processes. We define various forms of behavioral control as resulting from the interplay between three basic and computationally well-defined processes: signal detection, action selection, and action execution. Each process is monitored, and parameters are adjusted when the outcome is suboptimal. Furthermore, preparation will directly affect the effectiveness of the selection and execution processes. *Task rules*, which have to be activated and maintained, will constrain the processes and adjustments. Finally, we will outline how action control and behavioral change gradually becomes automatized through practice and, more generally, how a control system can develop.

## From Changes in the Environment to Changes in Behavior

**Fig. 2.**
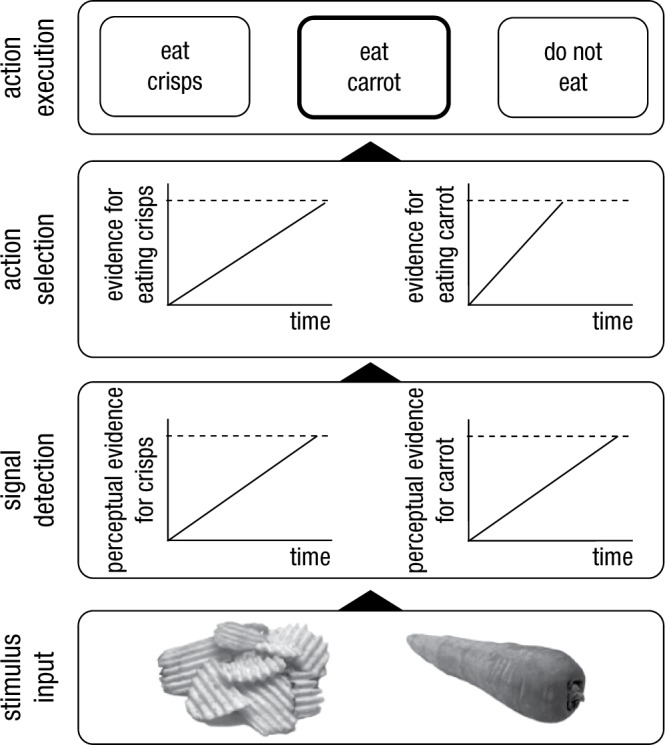
Action selection without a homunculus. We propose that action control involves three critical steps: signal detection, action selection, and action execution. We propose that both detection and selection can be modeled as accumulation of information toward a threshold (the dashed horizontal line). When stimuli are presented (in this example, the crisps and carrot), accumulation in the perceptual system starts, and a stimulus is perceptually encoded and attended (*signal detection*) when the evidence reaches a certain threshold. When an item is encoded, evidence for possible actions starts to accumulate (*action selection*), and a response is selected when one of the thresholds is reached. Then this response is executed. In this example, the “eat the carrot” threshold is reached first, so the person would eat the carrot. Note that for simplicity, we depict linear ballistic accumulators (S. D. Brown & Heathcote, 2008).

Flexible behavior is often studied in tasks such as the stop-signal paradigm (Logan, 1994; Verbruggen & Logan, 2008c), the psychological refractory period paradigm (Pashler, 1994; Welford, 1952), reversal learning paradigms (Izquierdo & Jentsch, 2012), Stroop tasks (Stroop, 1935), or in one of their many variants (e.g., Dodds, Morein-Zamir, & Robbins, 2011; Logan & Burkell, 1986; MacLeod, 1991; Mars, Piekema, Coles, Hulstijn, & Toni, 2007; Verbruggen, Aron, Stevens, & Chambers, 2010). These tasks often have in common that a new action has to be selected in the context of other strong action plans (see Table 1 for a selective overview of key paradigms). Differences in dependent variables such as response latency and error rates are usually assumed to reflect variations in the efficacy of control. For example, in most stop-signal task studies (including some of our own earlier work; e.g., Verbruggen, Liefooghe, & Vandierendonck, 2004), the stop-signal reaction time (Table 1) is assumed to reflect the duration of an executively controlled inhibition process. However, the stop-signal reaction time reflects more than the duration of an inhibitory process. Indeed, we have recently demonstrated how successful inhibition of actions depends on the detection of the stop signal and the selection of a stop response (Verbruggen et al., 2010; Verbruggen, Stevens, & Chambers, 2014). In this section, we will further develop our framework of how humans can change actions in various situations. Drawing on the seminal work of Sternberg (1969) and others, we propose that at the most basic cognitive level, action control involves three steps: signal detection, action selection, and action execution (their application is illustrated in Fig. 2).

**Table 1.** Overview of Popular Paradigms to Study Action Control and Behavioral Flexibility

Task name	Manipulation	Main dependent variable(s)
Stop-signal task	Participants usually perform a choice reaction time in which they have to respond as quickly as possible to a particular stimulus feature (e.g., color, shape, identity, or location). On a minority of the trials, the go stimulus is followed by an additional signal (e.g., an auditory tone or a visual cue), which instructs participants to withhold their planned response.	The stop-signal reaction time (SSRT), which is the estimated covert latency of stopping (Logan & Cowan, 1984). Longer SSRTs are usually interpreted to reflect poorer inhibitory control.
	In the countermanding task, participants have to cancel a saccade toward a target when a fixation cross reappears. In the stop-change variant, participants have to cancel the planned manual response and execute an alternative response instead.	
Go/no-go task	Participants are instructed to respond as quickly as possible to go stimuli (e.g., letters) but to refrain from responding when a no-go stimulus is presented (e.g., a digit). Go events typically occur with higher frequency than no-go events.	The probability of responding on a no-go trial.
Psychological refractory period (PRP) task	Participants are presented with two stimuli to which they have to respond. The interval between the two is usually so brief that the second stimulus appears before the response to the first one is finished.	Response latency of the second response (RT2), often as a function of the delay between the two stimuli (SOA). The PRP effect refers to the decrease in RT2 as SOA increases.
Stroop task and variants	In the Stroop task, color words are presented in various ink colors. Participants are instructed to respond to the ink color and ignore the words. In incongruent stimuli, color names and ink colors are non-matching. Related tasks include the picture-word naming task, in which words appear inside pictures of objects.	The congruency effect, which refers to the difference between incongruent and congruent or neutral (e.g., “OOO” written in red) stimuli.
The Eriksen flanker task	A task in which participants view target stimuli to which they must make a simple lexical response. These stimuli are surrounded by flankers. Distracting flankers are typically associated with an opposite response (*incongruent*), whereas facilitating flankers are typically associated with the same response as the target stimulus (*congruent*).	The congruency effect, which refers to the difference between incongruent and congruent items.
Task-switching paradigm	Participants frequently alternate between two or more tasks (e.g., naming the color or identifying the shape of a stimulus). Which task they have to perform is often indicated by a cue (e.g., the task name or the location of the stimulus) or by a sequence they have to remember.	The difference between task-switch trials and task-repeat trials. Usually, switching from one task to another is slower and more error-prone than repeating the same task.
(Wisconsin) Card Sorting Test	The participant is presented with stimulus cards containing shapes. The cards differ in color of the shapes, number of the shapes, and the form of the shapes. The participant is asked to sort these cards into two piles. The participant is not told what stimulus dimension to use in order to sort the cards, but feedback is provided to tell the participant if a particular match is correct. During the test, the sorting rules are changed and the participant must discover the new sorting rule in order to be successful.	The total number of categories achieved and the number of perseveration errors after a rule switch.
Response-reversal learning	Participants first learn to respond to stimuli based on feedback, followed by a reversal of the stimulus-action mapping. Participants have to overcome the old (habitual) response, and instead, execute an alternative novel response.	Proportion of correct responses before and after the reversal stage.

Note: Definitions are based on the Cognitive Atlas project (Poldrack et al., 2011). For more information about this project and other tasks, visit http://www.cognitiveatlas.org/. Note that this project also aims to increase the focus on the underlying processes. SOA = stimulus onset asynchrony.

### Signal Detection

The first step of replacing a response is nearly always detecting the stop or change cue (e.g., a traffic light turning red or noticing an unexpected obstacle on the road). A failure to detect the signal in time could have important negative consequences. However, the contribution of detection processes to executive control of action is often neglected.

A convergence of evidence suggests that flexible behavior depends on an interplay between two attention networks: a dorsal frontoparietal network that enables the selection of sensory stimuli and a ventral frontoparietal network that reorients attention to important and behaviorally relevant stimuli that appear outside the focus of attention (Corbetta, Patel, & Shulman, 2008; Corbetta & Shulman, 2002). The *dorsal attention network* is thought to be involved in both stimulus-driven capture (bottom-up) and goal-directed processing (top-down; Corbetta et al., 2008). Precisely how these modes interact is still intensely debated (e.g., see Theeuwes, 2010, and associated commentaries). The *ventral attention network* is believed to be critical for behavioral flexibility as it allows reorienting attention from one stimulus or task toward another. Even though this network is primarily involved in stimulus-driven attention, it is activated more by weak behaviorally relevant stimuli than by salient behaviorally irrelevant stimuli (Corbetta et al., 2008). This suggests that the detection of novel signals is constrained or biased by top-down control mechanisms. For example, target detection could be controlled by an *attentional template* (a representation of the relevant target or target features, such as a red circle) that biases competition between sensory inputs that compete for processing resources and control of behavior (Desimone & Duncan, 1995; Duncan, 2006). Computational work has shown how the templates could influence processing in both ventral and dorsal streams (Deco & Rolls, 2005). Preactivation of neurons in sensory areas that code for specific stimulus features (e.g., location, color, shape) could be the neural implementation of the templates (Deco & Rolls, 2005; Stokes & Duncan, 2013).

We believe that the literature on attention should become more integrated with the action-control literature. After all, signal detection is an important component of executive control of action: If changes in the environment are not detected quickly, this will have robust downstream effects on action control, as experimentally demonstrated in a recent stop-signal study (Verbruggen et al., 2014). Salinas and Stanford (2013) demonstrated that countermanding (i.e., canceling or stopping) eye movements primarily depends on the outcome of a rapid sensory detection process (see also Boucher, Palmeri, Logan, & Schall, 2007). On the basis of their computational work, they suggested that most manipulations in the countermanding task, which requires subjects to cancel eye movements (Table 1), cause changes in perceptual processes rather than inhibitory processes per se. The role of stimulus-detection or cue-encoding processes goes beyond action-reprogramming paradigms such as the countermanding task. Some models of interference control in paradigms such as the Eriksen flanker task (Table 1) focus on the role of spatial attention (e.g., J. D. Cohen, Servan-Schreiber, & McClelland, 1992). In the task-switching literature (Table 1), authors have argued that the cost associated with alternating between tasks is at least partly due to cue-encoding processes (Logan & Bundesen, 2003; Monsell & Mizon, 2006). Consistent with this idea, rule-switch performance in a card-sorting task in children was improved when the relevant stimulus dimensions were salient, suggesting a bottom-up attentional influence on flexible behavior (Fisher, 2011).

Thus, we suggest that rapid detection of cues or changes in the environment is key to replacing planned or ongoing actions and that at least some individual or situational differences in action control can be attributed to the efficacy of stimulus detection. Although signal detection may seem effortless, it does require a delicate balance between selective attention and change detection: Focusing on a given stimulus may lead to overly rigid behavior while the constant reorienting of attention to novel stimuli would lead to constant distraction (Verbruggen et al., 2014).

### Action Selection

When a change signal or cue is detected, an appropriate alternative action has to be selected. Sequential sampling models have provided a popular theoretical framework for action selection and decision making because they explicate the various steps involved (S. D. Brown & Heathcote, 2008; Ratcliff & Smith, 2004; Smith & Ratcliff, 2004). The main assumption of these models is that action selection and decision making involve the accumulation of noisy information about stimuli in the environment (Fig. 2). Noise is present both in the environment (e.g., visibility may be reduced or the environment may be cluttered) and in the cognitive system (e.g., neurons may fire randomly, and different processes may be happening at the same time), so decision making involves collecting evidence until there is enough support for an option. More formally, accumulation of information in response counters, which keep track of the collected evidence, starts when a stimulus is detected.^1^ In each situation, there may be different response options; one of them is selected when the accumulated evidence in favor of it reaches a certain threshold (Fig. 2). This response option is then executed. The more noisy the information (e.g., because the stimulus is perceptually degraded), the longer it will take to reach the threshold. This will result in longer reaction times and, often, lower accuracy. This accumulation to threshold may resemble patterns of activity in certain neurons (Purcell et al., 2010; X.-J. Wang, 2013). The main parameters of the selection process are the response criteria (i.e., how much information is required for a response to be selected; this is represented by the distance between the horizontal lines in Fig. 2) and accumulation rate (i.e., how quickly does the information accumulate; this is represented by the slope of the tilted lines in Fig. 2). Variations in these parameters can account for phenomena such as impulsive decision making and choice errors (Ratcliff & Smith, 2004; Smith & Ratcliff, 2004), and sequential sampling models have been successfully applied to a range of decision-making tasks and to different clinical and nonclinical populations (White, Ratcliff, Vasey, & McKoon, 2010).

These sequential sampling models have been applied mostly to simple decision-making tasks in which subjects have to select a single response. But we propose that similar principles underlie the selection of actions in the context of stopping, countermanding, or replacing actions. Sequential sampling models have already been applied successfully to countermanding (Boucher, Palmeri, Logan, & Schall, 2007; Salinas & Stanford, 2013) and stop-signal tasks (Logan, Van Zandt, Verbruggen, & Wagenmakers, 2014). Boucher et al.’s (2007) model included a single go accumulator and a single stop accumulator, with two processing stages, namely, encoding of the countermanding signal and interruption of the go process. An eye movement was successfully countermanded if stop information had accumulated quickly enough to suppress (via mutual inhibitory connections) information in the go unit and prevent it from reaching a threshold. Salinas and Stanford (2013) developed a similar rise-to-threshold model but did not make any mechanistic assumptions about inhibitory activity; as mentioned above, they showed that perceptual processes and deceleration of information accumulation was sufficient to account for many aspects of performance. These two models had only one go accumulator. More recently, a sampling model with multiple go accumulators and a single stop accumulator has been developed to account for stopping in situations in which multiple go responses are possible (Logan et al., 2014). This model could account well for both go (choice) and stop behavior.

In the context of behavioral change, alternative actions must often be selected in competition with more dominant or already activated actions. Existing models could easily be modified to account for this. It has been proposed that there may be an asymmetry in mutual inhibition of units (Boucher et al., 2007) or top-down biasing of response options (J. D. Cohen, Dunbar, & McClelland, 1990). More specifically, the mutual inhibition account assumes that different response options suppress each other. In Figure 2, when evidence for the carrot option accumulates, this would suppress accumulation of evidence for the crisps option. Boucher et al. (2007) suggested that there may be an asymmetry in mutual inhibition, so that one response option (e.g., the carrot) may suppress the other response option (e.g., the crisp) more. The biasing account assumes that information accumulation is biased (e.g., by decreasing the distance between the starting point and the threshold; see Fig. 2), making the selection of certain alternatives more likely. Others have proposed that selection of nondominant actions is achieved by global suppression of all motor output to allow for information accumulation in the counter of the nondominant action (Frank, 2006; Wiecki & Frank, 2013). The global suppression account overlaps with the “circuit breaker” account of attention, which proposes that when unexpected, salient signals are detected, ongoing processes are interrupted by default to allow the cognitive system to process the new incoming information (Corbetta et al., 2008; Corbetta & Shulman, 2002).

The computational work suggests that similar selection mechanisms may be used in various situations. Cognitive neuroscience studies further support this idea. Mid-dorsolateral and ventrolateral prefrontal areas are recruited by tasks that require selection of competing actions (Bunge, 2004; Duncan & Owen, 2000), and stimulation of these areas influences action selection in different situations. We have found that magnetic stimulation of subregions within the right ventrolateral prefrontal cortex can influence attentional and action selection not only in a stop-signal task but also in a double-response task in which participants occasionally had to execute a secondary response in addition to the originally planned response (Verbruggen et al., 2010). Similarly, Buch and colleagues demonstrated that stimulation of the ventral premotor cortex (adjacent to the posterior ventrolateral prefrontal cortex) influenced both selection and reprogramming of actions: Immediately after presentation of the initial stimulus, stimulation of this area increased motor cortex excitability; however, the same stimulation reduced motor excitability when reprogramming was required (Buch, Mars, Boorman, & Rushworth, 2010). This context-dependent effect of brain stimulation is consistent with the idea that similar mechanisms are involved in both programming and reprogramming actions, with the main difference being the output of the selection process (see also Mars et al., 2007). On the basis of these and similar findings, we propose that various forms of action control not only serve the common goal of behavioral change, they also rely on an overlapping set of selection processes (see also Mostofsky & Simmonds, 2008).

More generally, we propose that action selection and stimulus detection are governed by similar principles. The *biased competition* account of visual attention assumes that there is competition between sources of information at many (if not all) processing stages; the main role of attention is to resolve this competition by biasing specific sources of information or specific features (Desimone & Duncan, 1995). Similar top-down bias signals can resolve competition between action options or allow the selection of nondominant actions (see also Chun, Golomb, & Turk-Browne, 2011).

### Action Execution

When a response is selected or a decision has been made, the appropriate action must be executed. There can be a relatively long delay between choosing (or deciding) and acting (Schall, 2001) because a *motor program* has to be created when an action is selected. Keele defined a motor program as “a set of muscle commands that are structured before a movement sequence begins, and that allows the entire sequence to be carried out uninfluenced by peripheral feedback” (Keele, 1968, p. 387). Creating such programs after an action is selected may contribute to the delay between choosing and acting. Consequently, the execution phase could be a final stage where individual or situational differences in action control arise. Motor control is a research area in itself, and we will not attempt to review this literature here. We will focus briefly only on three topics that are closely linked to executive control, namely, the extent to which motor programs can be altered or canceled once initiated, how they are controlled after the action is executed, and how the motor system interacts with the cognitive system.

If a motor program contains all the information needed to carry out the action, no extra control is required to complete the desired movement. This does not imply that movements can no longer be altered. In the literature on action control, researchers often make a distinction between controlled stages, which could be influenced by executive control, and ballistic stages, which must run to completion once initiated. The boundary between these two stages is called the “point-of-no-return.” The stop-signal literature suggests that the ballistic stages must be very brief (Verbruggen & Logan, 2009b). This idea is supported by both mathematical analyses and studies that have showed that subjects could still inhibit responses that had already produced electrical activity in muscles (see Verbruggen & Logan, 2009b, for a review). Thus, movements can be prepared without being executed (Schall, 2001). Not only can planned movements be canceled, they can also be adjusted quickly if needed (e.g., Schmitz, Jenmalm, Ehrsson, & Forssberg, 2005). Thus, motor programs can still be terminated or altered quickly if new information becomes available.

Once the action has been completed, a “reset” signal may be required to restart evidence-accumulation processes and suppress motor activity to prevent the reexecution of the same response. Indeed, in many computational models, such a reset is required to stop the system settling into a loop. In Logan and Gordon’s executive control of theory of visual attention model, the executive system was responsible for this reset signal (Logan & Gordon, 2001). Note that there may be an overlap with the proactive suppression account discussed below, which proposes that people suppress motor activity to prevent premature responses.

In our framework, action execution is preceded by signal detection and action selection processes. This does not imply that action execution cannot interact with the preceding stages. First, an action can “create” a signal for the next decision. To achieve certain goals, multiple movements may be required. In some situations, these movements could be “chunked” or combined during the decision stage, when different options are selected simultaneously. But chunking may not always be possible (or preferable), so after each individual movement is completed, a new decision is required based on the imposed changes in the environment. This process would continue until the desired state or goal is achieved (G. A. Miller, Galanter, & Pribram, 1960). Second, the dual-task literature suggests that output modality has a direct influence on the decisional phase. Huestegge and Koch (2013) showed that saccades were prioritized over manual responses when participants had to make two decisions at the same time. This *response-modality dominance pattern* could be the equivalent of the *visual-modality dominance pattern* observed at the stimulus stage (i.e., visual modality often dominates the auditory modality when different stimuli are presented; see Huestegge & Koch, 2013). The combination of input and output modalities also influences task performance in dual-task situations (for a short review, see Huestegge & Hazeltine, 2011). For example, the dual-task cost, which is often observed in multitask situations (Table 1), disappears after sufficient practice for some input–output combinations (e.g., an auditory input and a vocal output) but not for others (e.g., visual input and vocal output). This suggests that selecting an action is influenced not only by the input but also by the output and the input–output pairing. More generally, this shows that there may be a close link between the cognitive and motor systems, which goes beyond the cognitive system instructing the motor system which actions to perform.

### Interim key points

- Researchers should provide a more detailed account of action control because the current focus on general functions hampers theoretical and practical progress.
- We propose a framework that describes three cognitive processes underlying most forms of action control: signal detection, action selection, and action execution. Each process can be conceived as a biased competition between alternatives.
- Individual or situational differences can arise at each stage, which further highlights the need for a detailed framework.

### Beyond online action control

We have outlined the core of our framework and have illustrated how replacing an action depends on the detection of change signals, selection of an action, and the execution of that action. The detection and selection stages involve a biased competition between sources of information and response alternatives. In the following sections, we will focus on how these biases are continuously adjusted by processes that take place across different timescales. We propose that detection, selection, and execution are influenced by monitoring, preparation, task rules maintained in memory, associative learning, and developmental changes (Fig. 1). Combined, these processes lead to flexible and highly adaptive behavior. In Figure 1, each component is depicted by a box. In the following sections, we will further unpack each box, creating our “army of idiots” (Monsell & Driver, 2000).

## Learning From Mistakes or Unexpected Outcomes

Many things can go wrong when people try to execute a novel action. People may confuse stimuli at the detection stage, they may select the incorrect response, or they may execute the selected response incorrectly. Even when no mistakes are made, the outcome of an action may be less desirable than anticipated. Monitoring and feedback loops are a critical component of most accounts of coordinated behavior (Ullsperger, Danielmeier, & Jocham, 2014). Within the executive control and decision-making literature, there are several detailed neurocomputational models of outcome monitoring that aim to explain how outcome monitoring influences subsequent detection, selection, and execution processes. Three popular classes of explanation are the conflict-monitoring, error-monitoring, and outcome-evaluation accounts.

### Conflict and error monitoring versus outcome evaluation

The conflict-monitoring account of Botvinick and colleagues (Botvinick, 2007; Botvinick, Braver, Barch, Carter, & Cohen, 2001) assumes that the anterior cingulate cortex, a brain area located in the medial frontal cortex, monitors for the occurrence of conflict between various response options. This brain region is often activated in situations in which prepotent responses have to be suppressed, situations in which one out of many possible but equally strong actions must be selected, situations in which errors are likely to occur, and situations with unfavorable outcomes. On the basis of these findings, Botvinick et al. (2001) proposed that the anterior cingulate cortex computes a “conflict signal.” Conflict can be defined computationally as the simultaneous activation of incompatible stimulus (Egner, 2008; Verbruggen, Notebaert, Liefooghe, & Vandierendonck, 2006) or response representations (Botvinick et al., 2001). When a conflict signal is generated, task-relevant attentional or action pathways are biased, reducing the likelihood of errors or conflict on subsequent trials (Botvinick, 2007; Botvinick et al., 2001). For example, Egner and Hirsch (2005) examined control adjustments in a picture–word Stroop task. On each trial, an irrelevant word was superimposed on a task-relevant face. They found that activation in the fusiform face area, a brain area that responds strongly to face stimuli, was increased after trials on which there was competition between the face and word stimuli. This is consistent with the idea that conflict-monitoring processes bias the competition between various sources of information, enhancing detection of task-relevant stimuli and selection of task-appropriate responses.

Others have stressed the role of anterior cingulate cortex in error-driven learning and computing the likelihood of errors (J. W. Brown & Braver, 2005). Brown and Braver showed how variability in a single error-learning parameter could account for individual differences in risk aversion and in the brain’s response to response conflict, error likelihood, or error consequences (J. W. Brown & Braver, 2008). Despite the differences, the conflict- and error-monitoring accounts stress that a critical aspect of optimal action control is the ability to monitor ongoing cognitive processes.

Outcome-evaluation models in the decision-making and reinforcement-learning literature assume that actions can be associated with a value, which represents a prediction concerning future reward. After every action, the cognitive system compares the obtained reward with the expected reward. After reward is obtained, the action values are updated: When the reward or outcome is better than expected, the strength of the selected action is strengthened (“reinforced”); when the outcome is worse than expected, the value is decreased, and the action is less likely to be selected again in similar future situations (e.g., Botvinick, Niv, & Barto, 2009; Frank & Badre, 2012). In other words, outcome-evaluation modulates action-selection biases, and this will influence how quickly information for the preferred response option will accumulate and reach the decision threshold. Some have argued that value can be attached to sources of information as well, influencing stimulus-detection processes (Gottlieb & Balan, 2010). Note that outcome evaluation and conflict and error monitoring could be two sides of the same coin (Botvinick, 2007). Indeed, conflict or choice errors could be construed as aversive events, which are therefore to be avoided in the future (Botvinick, 2007), and activation of the anterior cingulate cortex has been linked to encoding the relationship between an action and the reinforcement value of its outcome (Rushworth, Walton, Kennerley, & Bannerman, 2004, p. 412). Ridderinkhof, Ullsperger, Crone, and Nieuwenhuis (2004) suggested that a single mechanism that signals the likelihood of obtaining a reward could account for many findings in the reward-learning and conflict- or error-detection literature.

### Interim key points

- Adaptive behavior requires monitoring or evaluating the outcome of actions.
- Detection, selection, and execution biases are continuously adjusted as a result of the monitoring process. This will determine how quickly a stimulus is detected and how quickly an action is selected or executed in the future.

## Proactive Action Control

The work discussed so far may suggest that executive control is primarily reactive: It is only when something changes or when something goes wrong that the control system would kick in. However, in many situations, we do not wait for unexpected events to happen. Indeed, we can adjust our behavior proactively. In contrast to the online or reactive control processes discussed above, proactive control refers to control processes in anticipation of an event. Proactive control can involve many things, including preparing oneself to detect a stimulus or cue, activating specific action plans, temporarily adjusting decision thresholds, and suppressing motor output to prevent premature responses (Verbruggen & Logan, 2009c; Verbruggen et al, 2014). Thus, the three basic components of our framework may be influenced by preparation.

### Proactive adjustments of task settings

Humans can proactively allocate attention. For example, in the classic Posner cuing paradigm (Posner, 1980), detection of a stimulus is enhanced by providing a central cue (e.g., an arrow) that directs attention to a specific location (e.g., the left of the screen). This has been linked to anticipatory activity in the visual cortex (Kastner & Ungerleider, 2000; Luck, Chelazzi, Hillyard, & Desimone, 1997; Sylvester, Shulman, Jack, & Corbetta, 2007). Detection of stimuli or cues may also be enhanced by advance information of other features, such as shape, color, or direction of motion (Corbetta & Shulman, 2002). Nonspatial preparatory attention is also associated with sustained activity in sensory areas (Chelazzi, Duncan, Miller, & Desimone, 1998). Such an increase in baseline activity will lead to an increased probability that the system will select the stimulus that matches the attentional template. Thus, proactively adjusting attentional settings can enhance detection of task-relevant features (especially when perceptual information is weak) and reduce interference caused by no-longer-relevant features (Braver, Gray, & Burgess, 2007). Consistent with the latter idea, we have demonstrated that goal-directed cueing of the target location reduced the effect of distractors that flanked a target (Klemen, Verbruggen, Skelton, & Chambers, 2011).

Proactive action selection or movement preparation is also possible. For example, studies using a precuing procedure demonstrated that individual motor actions or sets of actions can be prepared or primed in advance (Rosenbaum, 1980; Rosenbaum & Kornblum, 1982). This could reduce the time required to create motor programs. Similar to attentional cueing effects, motor priming may be linked to anticipatory activation of the motor network via associations between the cue and action options. This will bias the selection and generally reduce the response time when a stimulus is presented (Meyer & Kieras, 1997). Consistent with this biased selection idea, computational modeling has shown that cuing the probability of a response or the potential payoff for a specific response reduces the amount of information required to select the more probable response or the response associated with higher reward (Mulder, Wagenmakers, Ratcliff, Boekel, & Forstmann, 2012). Priming of a nonhabitual response could also increase the probability of selecting this action in the context of other more habitual actions or when little information is available. Note that in some situations people may proactively suppress a specific action or set of actions to prevent the premature execution of a response (Cai, Oldenkamp, & Aron, 2011; Claffey, Sheldon, Stinear, Verbruggen, & Aron, 2010; Criaud, Wardak, Ben Hamed, Ballanger, & Boulinguez, 2012; Duque, Labruna, Verset, Olivier, & Ivry, 2012). Computational work by Lo and colleagues suggests that in a countermanding task, the stopping network is activated even when no stop signal is presented (Lo, Boucher, Paré, Schall, & Wang, 2009). Thus, inhibitory motor control in stop-signal and countermanding tasks may be largely proactive in nature because it depends on control adjustments and network dynamics before a stop signal is presented (see also X.-J. Wang, 2013, p. 238).

### Action control as a prepared reflex

Proactive control could potentially lead to a *prepared* or *intention-based reflex*. Some years ago, the second author was planning to turn into a road on his bike. A car was waiting to turn into the same road on the opposite side of the street the author was traveling along. The author had priority, as the car would cut across his path, and he made a clear signal with his extended arm just before he was about to turn. Unexpectedly, the car then immediately executed its maneuver, with the result that it knocked the author off his bike as he went around the corner. Why did this happen? We propose that when attention is proactively allocated and responses are prepared, goal-directed actions may not require much control anymore (Hommel, 2000; Logan, 1978; Meiran, Cole, & Braver, 2012); instead, actions could be activated easily by stimuli in the environment, even when they are inappropriate. Thus, when the car driver had prepared the response of turning to a high degree, the author’s signal with his arm may have further primed the prepared reflex to the point where it exceeded threshold and was implemented as an action.^2^

Logan (1978) demonstrated in a series of experiments that most stages in a visual-search task (including detection and response selection) remained relatively unaffected by a concurrent load. He concluded that the preparation before the stimulus appeared was effortful, but responding was reflexive: “the components of the task seem automatic, but the task itself is not” (Logan, 1978, p. 57). Similarly, Woodman, Luck, and Schall (2007) demonstrated that a visual working-memory load interfered with visual search only when the visual target changed from trial to trial. These findings suggest that stimulus detection, response selection, and execution may require little extra top-down support when correct task rules are properly activated. Furthermore, studies that have demonstrated that the preparation can even interfere with task-relevant or appropriate behavior (see also the bicycle anecdote) provide further support for the prepared reflex idea. Subjects are more likely to shift spatial attention to a task-irrelevant distractor when it matches a feature of the attentional template (Chun et al., 2011). Similarly, responses can be activated via instructed but unpracticed stimulus–response mappings even when these mappings are task irrelevant (Cohen-Kdoshay & Meiran, 2009); however, such effects are observed only when the tasks are actually prepared and participants anticipate that they have to perform them in the near future (Liefooghe, De Houwer, & Wenke, 2013). Finally, Verbruggen and Logan (2009a) found that the irrelevant distractor “STOP” inside a go stimulus interfered with responding in stop-signal and go/no-go tasks but not in a task where participants could always respond. (These findings are consistent with the prepared reflex idea: The prepared action can be triggered by irrelevant primes, even when this is not strictly required; Verbruggen & Logan, 2009a.)

### Costs of proactive control and individual differences

Combined, this work suggests that action control could be reflexive; paradoxically, this could even lead to a cost in some situations. But usually the main costs associated with proactive control are that this strategy requires highly reliable predictive contextual cues, it is metabolically costly, and it is capacity demanding (Braver et al., 2007). Humans usually prefer to avoid cognitive demands (Kool, McGuire, Rosen, & Botvinick, 2010), so internal costs may shift the balance between reactive and proactive control (McGuire & Botvinick, 2010). This also implies that a proactive strategy is less likely to be applied in situations with very long retention intervals between a warning cue and the stimulus, as this may require too much effort. Finally, strong preactivation of stimulus features or actions may also stand in the way of flexible behavior in ever-changing environments. Thus, a delicate balance between proactive and reactive control is required.

The costs associated with proactive control could potentially explain some individual and situational variation. Differences in motivation (Leotti & Wager, 2010) and emotional factors (Fröber & Dreisbach, 2012) contribute to intraindividual differences in deployment of proactive control, and factors such as reward sensitivity, general intelligence, and working-memory capacity may cause interindividual differences (Braver, 2012; Redick & Engle, 2011). Several studies have also shown group differences. Healthy young adults seem to rely more on proactive control than both young children (Munakata, Snyder, & Chatham, 2012) and older adults (Paxton, Barch, Racine, & Braver, 2008), and proactive control seems impaired in disorders such as schizophrenia, Alzheimer’s disease, ADHD, and bipolar disorder (for review, see Braver, 2012) and in individuals who engage in delinquent and antisocial behaviors (Iselin & Decoster, 2009). These findings suggest that at least some control deficits could be due to a failure to activate the control system proactively. One study revealed the interesting finding that training older adults on a proactive control task caused a shift from reactive to proactive control (Braver, Paxton, Locke, & Barch, 2009), suggesting that control strategies are amendable.

### Interim key points

- Executive control of actions is often proactive: The act of control takes place before the change or control signal is presented.
- When control is applied proactively, signal detection, action selection, and action execution can become a “prepared” reflex, easily triggered by information in the environment.
- Important intra- and interindividual differences could be due to shifts from proactive to reactive control.

## Activation and Maintenance of Action Goals and Rules

An important issue that we have not addressed so far is how the connections between input, action selection, and action execution are established. And how does the cognitive system “know” which stimulus feature or response option to bias? The main important advance of the (mathematical) modeling framework discussed above is that ongoing processes are described in detail. However, there is still a homunculus lurking: because it is the researcher who creates all the connections and sets up the relevant accumulators that enable a model to perform a certain task. Thus, this framework does not necessarily solve the problem of how the model could achieve this functionality in the first place. Most theoretical frameworks or models of executive control, including our framework, either explicitly or implicitly rely on rules (Bunge, 2004; Logan & Gordon, 2001; E. K. Miller & Cohen, 2001; Monsell & Driver, 2000). Rules enable humans to quickly select relevant cues or stimulus features, map sensory input to action plans, and produce the motor output. Furthermore, sequential adjustments after a trial (see, e.g., Hazeltine, Lightman, Schwarb, & Schumacher, 2011) and proactive control before a trial are also rule dependent. Thus, one could argue that rules are at the core of executive control. In this section, we will explore how rules are activated and maintained. We make an explicit distinction between a task goal and a task rule: A task goal describes what one tries to achieve, whereas a task rule specifies how one can achieve it. A goal will activate a rule (or set of rules). We will focus primarily on the role of task rules.

### Learning from instructions

A key characteristic of adaptive human behavior is the ability to rapidly learn action rules from instructions. For example, if instructed to tap your right foot whenever you see the symbol *x* on this page, most likely you will be able to do this without any practice (even though you have probably never done this specific task in your life). Recently, several studies have focused on the cognitive and neural mechanisms underlying this fundamental ability. For a complete overview of this instruction-based learning literature, we direct the interested reader to two recent review articles (Cole, Laurent, & Stocco, 2013; Wolfensteller & Ruge, 2012; see also Oberauer, 2009, who addresses the issue of language in rule learning and control). Cole et al. (2013) proposed the compositional account of instruction-based learning. Their account is based on five related principles: (a) *compositionality*, which refers to the ability to reuse representations with a variety of other representations; (b) *immediate transfer*, which refers to the ability to apply practiced rules to novel situations; (c) *abstraction*, which refers to the ability to group specific representations; (d) *analogy*, which refers to the ability to recognize similarities; and (e) *compositional hierarchy*, which refers to creating a structure in which more abstract representations modulate more concrete stimulus–action representations. Of these five principles, compositionality is key, as this can offer an elegant explanation for our remarkable ability to immediately perform tasks that we have never done before. Returning to the foot-tapping example, you may never have tapped your foot when you saw an *x* on a page, but you may have tapped your foot in response to other cues (e.g., music), and you may have searched for a specific word or letter string in a text before; by linking these representations, you are able to perform the new *x*-tapping task. In other words, you would reuse and recombine existing circuits, structures, or representations (see also Anderson, 2010). Support for the compositional theory and other relevant findings are discussed in Cole et al. (2013).

There are large individual differences in the ability to follow new task rules. The ability may be linked to fluid intelligence (Duncan, Schramm, Thompson, & Dumontheil, 2012). Furthermore, patients with lesions to the lateral prefrontal cortex may not always be able to produce the instructed behavior even though they can understand the instructions (Cole et al., 2013). Duncan and colleagues (Duncan, Emslie, Williams, Johnson, & Freer, 1996; Duncan, Johnson, Swales, & Freer, 1997) have referred to this phenomenon as *goal neglect*. Verbal instructions specify an abstract requirement (e.g., “*if x*, then tap right foot”), but these requirements have to be implemented or transferred to procedural working memory (Duncan et al., 2012; Logan & Gordon, 2001; Oberauer, 2009). For example, relevant stimulus information, response options, and output modalities should become biased, and contexts in which the rules are relevant specified. A failure to do so would lead to goal neglect (Duncan et al., 1996; Duncan et al., 1997).

### Maintenance and retrieval of task rules

When instructions are successfully implemented, rules have to be maintained. We have argued above that there is sustained activity in brain areas that process task-relevant information, which biases the selection of information. Rules likely provide the top-down signal for this bias (E. K. Miller & Cohen, 2001; Stokes & Duncan, 2013). The popular account is that rules are maintained in working memory via persistent firing of stimulus-specific neurons in the prefrontal cortex (Curtis & D’Esposito, 2003). More generally, this persistent firing would allow temporal integration of information, which is required for many functions, including working memory and the calculation of reward rate (Curtis & Lee, 2010). However, recent findings challenge this *persistent activation* account (Postle, 2013; Stokes & Duncan, 2013). For example, Stokes and colleagues (2013) showed that the presentation of an instruction cue triggers a sequence of high-activity patterns before settling into a stable low-activity state. They proposed that, rather than sustained activity, synaptic weight changes constitute the task-dependent rules that determine how people respond to stimuli (Stokes & Duncan, 2013). One of the main challenges is to further determine how rules are maintained in long-term and short-term memory.

In many situations, people also have to switch between rules. This fundamental ability is studied in the task-switching paradigm (for reviews, see Kiesel et al., 2010; Monsell, 2003; Vandierendonck, Liefooghe, & Verbruggen, 2010). Switching from one rule to another is usually associated with a performance cost. Most agree that this switch cost reflects the time needed to encode the task cues, activate the appropriate task rules, and resolve interference caused by previous trials, although the extent to which each process contributes to the overall switch cost may vary. Cue encoding and task reconfiguration are time-consuming processes, so performance generally improves when these processes can be completed before the stimulus appears (Logan & Gordon, 2001; Mayr & Kliegl, 2000; Meiran, 1996; Rogers & Monsell, 1995). This demonstrates the close link between preparation and rule activation and maintenance. However, not everybody agrees that people always have to switch or update rules when tasks change. Logan and colleagues argued that switching between tasks could be achieved via the retrieval of learned associations among cues, stimuli, and responses (Logan & Bundesen, 2003; Schneider & Logan, 2005), although this idea remains highly controversial (e.g. Forrest, Elchlepp, Monsell, & McLaren, 2012; Jost, Mayr, & Rösler, 2008; Monsell & Mizon, 2006). In other words, they argued that “control” could be associatively mediated.

### Interim key points

- In our framework, rules constrain performance by providing a top-down bias for each process.
- We argue that the ability to follow novel instructions and implement new rules is strongly rooted in the past: Humans constantly reuse and recombine old rules that have previously governed behavior.

## Action Control as an Associatively Learned Reflex

Historically, executive control has been pitted against automatic operations. Often, functions such as response inhibition, interference control, or task switching have been classified as “executive,” whereas other processes, such as word reading in the context of a Stroop task, have been classified as “automatic.” In this section, we discuss how executive processes may also become automatic as a consequence of practice.

### Automaticity and associative learning

It is well documented that responding to a stimulus or cue can become automatized over practice (Dickinson, 1985; Logan, 1988; Schneider & Shiffrin, 1977; Shiffrin & Schneider, 1977). Shiffrin and Schneider proposed that when a stimulus and a response are consistently mapped, associations are formed, allowing actions to become automatic (Schneider & Shiffrin, 1977; Shiffrin & Schneider, 1977). Similarly, Logan (1988) suggested that every time people respond to a stimulus, processing episodes are stored. These episodes consist of the stimulus (e.g., “3”), the interpretation given to a stimulus (e.g., “odd”), the task goal (“odd/even task”), and the response (“left”), all of which are stored. When the stimulus is repeated, previous processing episodes are retrieved, facilitating performance if the stimulus–response (S-R) mapping is consistent. Recently, researchers from our team have demonstrated that more complex forms of action control could also become automatized. We found that pairing a stimulus with stopping interfered with responding to these stimuli (Verbruggen & Logan, 2008a), even after a single stop presentation (Verbruggen & Logan, 2008b; Verbruggen, Logan, Liefooghe, & Vandierendonck, 2008). We attributed the behavioral slowing for old stop items to the retrieval of stimulus–stop associations, which would automatically suppress responding. Similar associatively mediated “control” effects have been observed in other executive control tasks. The task-switching literature suggests that stimuli can become associated with tasks or rules (Koch & Allport, 2006; Mayr & Bryck, 2005; Waszak, Hommel, & Allport, 2003). For example, the results of Mayr and Bryck (2005) suggested that abstract spatial translation rules can become integrated with lower level stimulus and response codes; similarly, the results of Waszak and colleagues suggested that individual stimuli can become associated with higher order task representations, such as picture naming (Waszak et al., 2003). Finally, several studies have shown that stimuli in tasks such as the Stroop paradigm can become associated with attentional control settings (Bugg & Crump, 2012). On the basis of these findings, we argue that rule-based action control can also become a learned reflex, triggered even when it is not required or intended at a given moment (cf. Meiran et al., 2012; Tzelgov, 1997; Verbruggen & Logan, 2009a)

### Associative influences on action control

We suggest that there are four nonmutually exclusive ways that associative learning could influence action control: (a) conditioned attention toward (or away from) the cues, (b) associatively mediated activation of previously nondominant responses, (c) associatively mediated activation of abstract rule representations, and (d) by changing the hedonic and/or incentive value of stimuli.

First, associative learning could influence attentional selection. For example, Le Pelley, Beesley, and Griffiths (2011) have found that subjects looked more at cues experienced as predictive of the outcomes with which they were paired than to cues experienced as nonpredictive. Similarly, Gottlieb and Balan (2010) reviewed a single-cell recording study that showed higher sustained lateral intraparietal area activation for predictive cues in a Pavlovian task and suggested that attentional selection is influenced by the information value of the stimuli. These results are consistent with the attentional model of associative learning proposed by Mackintosh (Mackintosh, 1975; Pearce & Mackintosh, 2010). However, Hogarth and colleagues (Hogarth, Dickinson, Austin, Brown, & Duka, 2008) have found that participants looked more at partially predictive signals in some situations, which is consistent with the Pearce–Hall model of Pavlovian learning (Pearce & Hall, 1980; Pearce & Mackintosh, 2010). Even though there is uncertainty about the direction of the effects, it is clear that attention and associative learning can interact (albeit in various ways), and the model of Mackintosh and Pearce integrates earlier accounts to reflect this (Pearce & Mackintosh, 2010). In other words, attention can become conditioned (McLaren, Wills, & Graham, 2010): Attention is paid to stimuli as a consequence of past associative history, rather than because of their match with current goals. This is also supported by some event-related potential (ERP) studies. For example, Wills and colleagues have demonstrated that early attentional components were modulated by associative learning (Wills, Lavric, Croft, & Hodgson, 2007). In a similar vein, ERP work reviewed by Woodman suggests that top-down biasing of visual attention is required only when targets are new, with long-term memory taking over when objects are repeated (Woodman, 2013).

Second, a nonhabitual response could become habitual after sufficient practice (Dickinson, 1985; Logan, 1988; Schneider & Shiffrin, 1977; Shiffrin & Schneider, 1977). This would further reduce the need for top-down biasing; after sufficient practice, the need for top-down biasing may even disappear altogether, and people would no longer have to rely on rules or prefrontal cortex (PFC) representations to execute an action that was initially nondominant. This idea is supported by the work discussed above and by the finding that neural activation in prefrontal and other control-related brain regions is reduced after practice with consistent mappings (Chein & Schneider, 2005).

Third, the studies discussed above suggest that during practice, stimuli can become associated with task goals or the task rules that bias attentional or action selection. After practice, the goal or rule representations may become activated when a stimulus is repeated, whether this is intended or not; this would then influence subordinate processing. The stimulus-rule association idea could explain why repeating an old stop stimulus activates components of the stopping network in the ventrolateral prefrontal cortex (Lenartowicz, Verbruggen, Logan, & Poldrack, 2011) or why naming the word inside a picture–word Stroop stimulus is impaired when this stimulus was previously encountered in a picture-naming task (Waszak et al., 2003). Note that such stimulus-task effects were observed even when the response (e.g., a left key press) was the same in both tasks (Koch & Allport, 2006; Waszak et al., 2003). The main difference from the previous two options is that this third option assumes that rule-like representations that bias ongoing selection processes are still involved. In other words, this third option provides a more indirect route to associative control of action. However, a possible advantage of this route is that this form of learning might generalize better to novel situations. We are currently testing this idea in our lab. Note that in the associative-learning literature, there is an ongoing debate as to whether learning associations between a stimulus and an action is based on rules or on the formation of specific S-R associations (see, e.g., McLaren et al., 2013; Mitchell, De Houwer, & Lovibond, 2009). Even though this is speculative, one could hypothesize that in our framework, similar learning mechanisms underlie rule-based behavior and S-R link-based behavior. The main difference between the two is the kind of representation that is linked with the stimulus: an abstract, rule-like representation (*x*–“if *x*, then left”), or more concrete S-R associations (*x*–left).

Finally, stimulus-specific learning may also have a more indirect impact on action control via a link with the outcome-evaluation processes discussed above. Veling, Holland, and van Knippenberg (2008) have shown that consistently pairing stimuli with the act of withholding a response results in devaluation of stimuli that were initially rated as positive. Similar devaluation effects have been observed in other studies (for a short overview, see Ferrey, Frischen, & Fenske, 2012; Kiss, Raymond, Westoby, Nobre, & Eimer, 2008). Furthermore, no-go training cannot only reduce the subjective hedonic value of erotic images; it may also reduce the motivational incentive of such stimuli. Ferrey et al. (2012) found that participants were less willing to invest time and effort (measured by the number of key presses participants were willing to execute) to view images similar to the ones paired with no-go cues. The link between associative learning and value is also supported by research in the animal learning literature (for a recent overview, see McLaren & Verbruggen, in press). Furthermore, learning stimulus–go associations happens faster in a reward condition than in a punishment condition; by contrast, stimulus/no-go associations are learned faster in the punishment condition (Cavanagh, Eisenberg, Guitart-Masip, Huys, & Frank, 2013; Guitart-Masip et al., 2012). Thus, there may be a “hardwired” Pavlovian bias that couples reward with approach (“go”) and punishment with avoidance (“no-go”) (Cavanagh et al., 2013; Guitart-Masip et al., 2012). Note that the value of items could also be modulated associatively via associations between stimuli. Wimmer and Shohamy (2012) demonstrated that the delivery of reward for a specific item can spread to associated items stored in long-term memory. In other words, the value of unrewarded items was modulated via associations with rewarded items. This phenomenon could explain how people can quickly decide between items that they have never seen before.

In combination, the work above suggests how changing behavior can become automatized. However, work on extinction learning indicates that some associatively mediated forms of action control may be context dependent (in contrast to rule-based action control, which seems context independent). In Pavlovian learning, extinction occurs when a stimulus that was originally paired with an event is repeatedly presented alone; in instrumental learning, extinction occurs when an action that was originally paired with a reward is no longer reinforced. In both cases, learned behavior typically declines, but the originally learned behavior often returns when the context changes (Bouton & Woods, 2008). This suggests that extinction learning is context dependent. Thus, even though automatization may lead to more efficient action control, it does come with certain limitations.

### Interim key points

- Action control can become a “learned” reflex: Replacing dominant actions initially requires top-down bias but could gradually become automatized, with the need for top-down bias disappearing altogether.
- Associative learning can influence action control by modulating each processing step in our framework (i.e., signal detection, action selection, action execution).
- We hypothesize that similar learning mechanisms underlie both rule-based and S-R link-based behavior.

## Development of an Action Control Network

We argued above that a key characteristic of flexible human behavior is the ability to implement new rules quickly, and we proposed that this feat can be achieved by reusing or recombining existing representations or rules (i.e., the compositionality idea). But in our quest to abolish the control homunculus from theories of action control, we need to address one final issue: How does the *control repertoire*, or the set of basic rules and control processes, initially develop?

### Learning of rules and abstract representations

Little research has been done on how rules for complex actions are initially acquired (Collins & Frank, 2013). Basic reinforcement learning accounts can explain how people acquire simple stimulus–action rules. A central assumption of these accounts is that simple rules are learned via exploration of the environment: When a stimulus is presented, one can try different courses of action (e.g., pressing a button on a new piece of equipment) and subsequently monitor the outcome of the chosen actions. Each time a particular action in response to the presentation of a stimulus leads to a positive outcome, the strength of the action increases, and eventually, simple rule-like structures develop. As argued above, stimuli and responses can also become paired via error-driven and Hebbian associative learning mechanisms. Error-correction learning mechanisms try to reduce the discrepancy between the predicted outcome and the actual outcome (McLaren et al., 2013), whereas Hebb’s learning rule states that “cells that fire together bind together.” However, basic reinforcement and associative learning accounts struggle to explain more complex goal-directed behavior in environments in which multiple stimuli or stimulus features (e.g., color or shape) can be attended to and in which many actions can be selected.

One of the harder questions in psychology is how, starting with a set of basic associative- or reinforcement-learning processes, it might be possible to deploy them so as to arrive at a system capable of propositional reasoning. In other words, how can we go from associations to rules? There have been some successful attempts to integrate basic learning and rule acquisition. For example, Rougier, Noelle, Braver, Cohen, and O’Reilly (2005) developed a neurologically inspired computational model of rule learning. The model was trained to respond to multidimensional stimuli. In each block, only one dimension was relevant (e.g., color). Across trials, the specific features within a dimension changed (e.g., red, green, yellow), but activity in the prefrontal cortex was maintained because of a gating mechanism (Hazy, Frank, & O’Reilly, 2007). As a result, the PFC system developed patterns of activity that encoded abstract representations of the relevant stimulus dimension (e.g., “color”). These abstract rule-like representations subsequently guided behavior by providing “top-down” excitatory support for the relevant stimulus dimension in the subordinate processing levels (cf. biased competition). The biasing was possible because links between the abstract representations and the processing layers were built during training. Thus, the model produced flexible rule-like behavior without “biologically problematic symbolic processing computations” (Rougier et al., 2005, p. 7343). After sufficient training, the model was also able to respond correctly to stimuli it had not seen before. This generalization correlated strongly with development of abstract representations. By contrast, models without the PFC system, such as the associative simple recurrent network model (Elman, 1990), had to learn specific S-R combinations, and these did not generalize well to novel situations.

The latter result is consistent with the findings of Spiegel and McLaren (2006). Humans and the recurrent network model were trained on a task in which they had to respond to the location of circles on a computer screen. In the experimental group, trial sequences always had a specific structure (e.g., ABB[varying numbers of Cs]BBA; the letters represent three possible locations of the circles). Over a series of experiments, it was demonstrated that the network model used all the structure available to develop simple rule-like representations. This resulted in faster and more accurate responses. These representations allowed some generalization to novel situations (hence, they were rule-like). However, generalization was imperfect because the model was sensitive to seemingly inconsequential departures from the initial structure. This was very similar to the behavior of humans in these experiments when they were unaware of the contingencies in play. However, under some conditions, humans were able to induce the rule as programmed by the experimenters (e.g., “always as many Bs before as after the Cs”), and in these instances generalization was near perfect. The recurrent network model was never able to do this. This suggests that rule learning in humans comes about as the result of a more complex system with many interacting parts. Nevertheless, the finding that a simple associative network is able to develop basic rules (albeit imperfectly) further supports the idea that basic associative or reinforcement mechanisms may indeed underlie rule learning. Consistent with this, Ramamoorthy and Verguts (2012) recently developed a computational model of instruction following that relied on basic Hebbian learning processes in prefrontal cortex (see also Deco & Rolls, 2005).

If we assume that we begin with reward- or error-driven associative learning processes, then we believe that in order to be capable of developing rule-like representations, these processes will need to be embedded in an architecture that must, at a minimum, be complex (by which we mean multilayer or more than one layer of weights), nonlinear (so that the multiple layers are not simply equivalent to a single layer; see Minsky & Parert, 1969), and recursive (so that the system can, in principle, compute any computable function). Obviously, the learning algorithm used will have to be capable of operating within this framework (for an example of such an algorithm, see Rumelhart, Hinton, & Williams, 1986). In essence, then, we propose that associative processes within a sufficiently rich and complex architecture can deliver the possibility of rule-based (symbolic) computation. But even if all these requirements are met, there is still much to be done. It will be the interaction of that system with the world that will allow this development to take place. The architecture and learning algorithms, which have evolved throughout human evolution, merely confer the potential for rule-based processing; the potential has to be realized in the course of experience, and so the transition from association to cognition is also a developmental issue.

In sum, we believe that a key to behavioral flexibility is the development of abstract representations via basic learning mechanisms. These representations will guide or contextualize stimulus detection, action selection, and action execution (Badre, Kayser, & D’Esposito, 2010; Collins & Frank, 2013; see also, e.g., Frank & Badre, 2012) and allow generalization to novel situations (see also Forrest et al., 2012). Even though these models were used to simulate relatively straightforward rule-based behavior, the general principles are likely to apply to more complex situations (Rougier et al., 2005). In complex environments, the agent may make temporal abstractions: grouping together a set of interrelated actions (*options* or *policies*) (Botvinick, 2012; Botvinick et al., 2009). These policies can be learned and selected via the same reinforcement-based learning mechanisms discussed above. When a policy is selected (e.g., making coffee), the more “primitive” motor actions are produced based on the acquired lower level stimulus–action associations (see Botvinick, 2012, for an accessible discussion).

### Development: Building a network for the future

Major changes in rule-based action control take place during childhood. Indeed, the ability of children to regulate their behavior improves remarkably from infancy through adolescence (for recent reviews, see Bunge & Crone, 2009; Bunge, Mackey, & Whitaker, 2009; Diamond, 2013). Such changes have been linked to development of executive control functions and the protracted development of the prefrontal control network (Bunge & O’Hare, 2012).

Developmental changes in rule-based action control throughout early and late development can be linked to a shift from concrete stimulus–action associations to abstract rule-like representations (Bunge & Zelazo, 2006; Munakata et al., 2012). Initially, young children would learn simple stimulus–action associations via exploration (“if I push this button, a light turns on”), automatic imitation, or deliberate demonstration by others. These associations then become the building blocks for the rule-based control network and shape the development of more abstract representations that constrain and regulate other ongoing processes. Indeed, Rougier et al. (2005) found that concrete S-R representations (in posterior brain systems) had to stabilize before abstract rule-like representations could be extracted.

Several studies support the transition account. For example, young children are influenced more by specific S-R associations than adults when switching between tasks (Crone, Bunge, van der Molen, & Ridderinkhof, 2006). Furthermore, Kharitonova and Munakata (2011) have demonstrated that in 3-year-old children, flexible rule use in a card-sorting test correlated with performance in an abstraction test that required children to select the odd-one-out on the basis of an overarching category. They suggested that this link could be explained by a common underlying working memory mechanism that supports rule-like abstraction and perceptually based abstraction (see also Collins & Frank, 2013). In other words, abstraction underlies flexibility.

In addition to changes in the ability to develop abstract rule-like representations, children may also develop an ability to generate temporal abstractions. Botvinick et al. (2009) noted that throughout development, action control becomes more hierarchical, with simple actions or rules becoming integrated within larger wholes or structures. Similarly, Bunge and Zelazo (2006) reviewed a series of studies suggesting that development of cognitive control was associated with an increased ability to represent hierarchies of rules in which higher-order rules (cf. “policies”) operate on lower order rules.

To conclude, Thompson-Schill, Ramscar, and Chrysikou (2009) proposed that protracted development of the executive prefrontal network is necessary to allow children to learn linguistic and social conventions. Here we propose that learning necessarily precedes executive control because learning has to provide the building blocks for a control repertoire based on abstraction first.

### Interim key points

- Our framework places learning of increasingly abstract representations at the heart of executive control.
- Only through constant interaction with their environment can people build up a control repertoire that will underlie all forms of rule-based behavior.
- This repertoire continues to develop throughout the life span.

## Implications for Behavioral Change

Clinical disorders often have many origins; alterations of cognitive processes may be one of them. Therefore, we believe that our framework can be applied in clinical domains to increase our understanding of certain control deficits and provide a theoretical basis for the development of novel behavioral change interventions.

Just as in the cognitive and neuroscience domain, attribution of control to convenient control homunculi is still very present in the clinical and more applied domains. Most clinical and neurobiological models that rely on executive control lack a precise description of the underlying cognitive components and mechanisms. We have argued that a failure to change behavior could have multiple origins. Thus, merely describing a deficit or phenomenon as a deficit of “inhibition” or “executive control” does not provide an explanation and discourages discussion of alternative theories. For instance, poor stopping performance in adults with ADHD may be partly due to inattention (Bekker et al., 2005). Many studies have observed stopping deficits in children and adults with ADHD, which has led researchers to suggest that poor response inhibition is central to their deficit (Lijffijt, Kenemans, Verbaten, & van Engeland, 2005; Lipszyc & Schachar, 2010; Nigg, 2001). However, Bekker et al. (2005) found using ERPs that an early attention-related component (the N1, which is a negative-going ERP component observed 80–120 ms after the presentation of an auditory stop signal) was larger for successful stop trials than for unsuccessful stop trials in the control group. This finding suggests that perceptual attention contributes to stopping. This difference in N1 was absent in adults with ADHD, which suggests that stopping deficits in adults with ADHD are due not entirely to deficiencies in inhibition but also to deficiencies in stimulus detection. Similarly, J. W. Brown and Braver (2008) have argued that the failure to suppress risky and inappropriate behavior in addictions could stem from a failure to adjust performance after learning (for a similar idea, see Garavan & Stout, 2005). These studies indicate that focusing on basic processes provides a more detailed account of control deficits in, for example, behavioral and substance addictions. This may lead not only to important new insights in the etiology of various disorders characterized as deficits in changing behavior but also to the development of strategies for treating these conditions. Indeed, a common critique is that the effective mechanisms of most behavioral treatments are still underspecified (Toneatto & Ladoceur, 2003). Therefore, providing a detailed account of action control deficits could stimulate the development of new theory-driven behavioral treatments. For example, it could lead to personalized interventions: Person A and Person B may both have stopping deficits with different origins, so the intervention for Person A could, for example, focus on biasing attention (e.g., in adults with ADHD; see Bekker et al., 2005), whereas the intervention for Person B could focus on performance monitoring and control adjustments (e.g., in substance abusers; see Garavan & Stout, 2005).

The work on proactive control suggests that preparation could lead to a prepared reflex, making action control less susceptible to the negative effects of concurrent load (Logan, 1978). This is consistent with findings in the *implementation intention* literature (Gollwitzer, 1999; Gollwitzer, Gawrilow, & Oettingen, 2010; Gollwitzer & Sheeran, 2006). Implementation intentions refer to the linking of critical situations or cues to specific actions (e.g., “Whenever I see a red light on a food item, I will not buy it”). This could lead to a prepared reflex; indeed, Gollwitzer noted that after implementation intentions are formed, “action initiation becomes swift, efficient, and does not require conscious intent” (Gollwitzer, 1999, p. 495). Others have argued that forming implementation intentions leads to increased monitoring for cues (see Rummel, Einstein, & Rampey, 2012, for a discussion), but this is still consistent with the proactive control idea discussed above. It is important to note that implementation intentions, and consequently proactive control, could have a positive impact on behavior (Gollwitzer et al., 2010; Gollwitzer & Sheeran, 2006). For example, they may reduce the negative impact of stress on rule-based action control (Scholz et al., 2009), presumably because less reactive control is required. They may also strengthen the effects of commercial weight loss programs (Luszczynska, Sobczyk, & Abraham, 2007) and reduce binge drinking (Hagger et al., 2012). Thus, an avenue for future research is how proactive control can be used in treatments, bearing in mind that there are certain costs associated with it (as discussed above).

It is also important to understand how people develop and use new rules. For example, supermarkets in the United Kingdom recently started using a traffic-light labeling system to indicate sugar, fat, salt, and calorie contents of food items. But how do people use this new system to replace their favorite (but unhealthy) food item with a more healthy option? The work on rule learning and, in particular, generalization and abstraction as discussed above could provide some clues. For example, it suggests that new rules that are based on previously acquired rules (e.g., red light = stop) might be learned more quickly (and consequently be more effective). The ability to form abstract rules may also lead to generalization of control across domains. We agree with Munakata et al. (2012), among others, that abstraction may explain executive-control training effects in children. Executive-control training may work better in children with low self-control than in adults with low self-control (Berkman, Graham, & Fisher, 2012). A better understanding of how rules are developed would lead to more effective training. Even though this is highly speculative, building abstract rule-like representations may also provide an explanation for some more idiosyncratic transfer effects, such as the positive effect of avoiding sweets or regularly squeezing a handgrip in a 2-week training period on stop performance afterward (Muraven, 2010), the differential effect of open versus skilled sports on stopping (C. H. Wang et al., 2013), or other inhibitory spillover effects (e.g., Berkman, Burklund, & Lieberman, 2009). As proposed by the compositional account, people may recycle or recombine rules that they used in other situations; building up a control repertoire in one domain could therefore lead to improved performance in other apparently nonrelated domains as long as the acquired representations are abstract enough.

Finally, associatively mediated action control could open the avenue for new treatments. Several recent studies have already shown that learning to stop motor responses toward food- or alcohol-related stimuli influences food and alcohol intake both inside and outside the lab. For example, several studies have found that consistent pairing of food-related pictures to stopping in a go/no-go or stop-signal paradigm reduced subsequent food consumption (Houben, 2011; Houben & Jansen, 2011; Lawrence, Verbruggen, Adams, & Chambers, 2014; Veling, Aarts, & Papies, 2011; Veling, Aarts, & Stroebe, 2012). Furthermore, a similar procedure with alcohol-related stimuli reduced the hedonic value of alcohol and the subsequent weekly alcohol intake of heavy drinking students (Houben, Havermans, Nederkoorn, & Jansen, 2012), whereas Jones and Field found that stimulus-specific stop training reduced alcohol intake in the laboratory but not self-reported drinking in the week after training (Jones & Field, 2013). Wiers and colleagues found that a similar avoidance training task influenced treatment outcomes in alcoholics a year later (Wiers, Eberl, Rinck, Becker, & Lindenmeyer, 2011). Finally, recent work from our lab suggests that stopping a motor response can reduce gambling (Verbruggen, Adams, & Chambers, 2012). We are currently exploring the mechanisms behind this transfer, but it is possible that stopping generally reduced approach motivation. In combination, these results suggest that go/no-go, avoidance, or stop-signal training can influence approach behavior toward a range of stimuli, possibly by changing attitudes toward these stimuli or by creating nonrespond (avoid) associations. People may also associatively learn to direct their attention either toward or away from stimuli. Recent meta-analyses suggest that attentional-bias modification could be used in treatments for anxiety (Hakamata et al., 2010), although the effect may be more modest than initially suggested (Hallion & Ruscio, 2011). Several studies have also examined attentional-bias modification in addiction. This could involve training people to redirect attention away from drug-related cues toward more neutral cues (Wiers, Gladwin, Hofmann, Salemink, & Ridderinkhof, 2013). The effectiveness of this training on addiction is still unclear. For example, a single session of attentional bias modification did not influence subjective craving or behavioral measures of tobacco seeking in cigarette smokers (Field, Duka, Tyler, & Schoenmakers, 2009). Approach–avoidance, go/no-go, or stop-signal training may be more effective because several aspects of inappropriate behavior can be influenced at the same time. Indeed, avoidance or inhibition training could influence hedonistic value, motivational behavior (approach vs. avoidance), and possibly even attention toward the stimuli. Data from studies inspired by the framework presented here suggest that, in some situations, subjects may learn associations between the go stimulus and the stop signal, enhancing detection of the latter (Verbruggen, Best, Bowditch, Stevens, & McLaren, 2014). However, much more research is needed to examine how well various forms of inhibitory and executive-control training can influence behavior outside the lab (see also Jones, Christiansen, Nederkoorn, Houben, & Field, 2013). This work will also have to address the context-dependence issue (Bouton & Woods, 2008).

## Final Thoughts and Conclusions

We have discussed how action control can be attributed to the interplay between three basic cognitive processes: signal detection, action selection, and action execution. These processes are constantly adjusted and biased via abstract representations that develop slowly but that can be generalized to different contexts. These representations support flexible behavior. At the same time, more concrete stimulus–action outcome associations are learned, which can result in automatization of actions that were initially regulated by the “executive” abstraction-based system. We have attempted to unpack each component of our framework. It is possible that future research will demonstrate that some components or processes may have to be broken down further, leading to an even more nested system. We agree with Dennett that the only way to “discharge fancy homunculi from one’s scheme [is] by organizing armies of such idiots to do the work” (quoted in Monsell & Driver, 2000, p. 7). We believe that in order to understand how control is achieved, boxes have to be broken down until we understand how complex behavior arises from a basic set of cognitive processes that can be implemented by our neural system. One may object against this deconstruction idea on the basis of parsimony: A model with fewer components may seem more parsimonious. However, this parsimony would be achieved only by attributing multiple powers to specific controllers or control functions (Monsell & Driver, 2000), making the seemingly more parsimonious account equally complex.

### Relation with other frameworks and models

Our framework builds on existing accounts of attention, control, working memory, and learning (e.g., Chein & Schneider, 2012; Deco & Rolls, 2005; Desimone & Duncan, 1995; Logan & Gordon, 2001; E. K. Miller & Cohen, 2001; Rougier et al., 2005). Consistent with these accounts, we postulate that the main role of the executive control system is to bias competition in subordinate processes via rules maintained in working memory (either in an active or silent mode). But we also propose that once the rules are implemented, the control system can take a back seat in many, if not most, situations, and action control may eventually become automatized. This overall framework is consistent with the *learning and control* framework of Chein and Schneider (2012), who proposed that there are three systems: a *meta-cognitive system*, which supports rule learning, monitoring, and task sequencing (cf. hierarchical control); a *cognitive control system*, which supports attention and action control; and *a representation system*, which supports associative learning. They also suggested that through practice, the associative system will take over from the metacognitive and control systems, which is consistent with our “learned reflex” idea.

We believe that the main strength of our framework is that it integrates various theories and models, links findings, and points out similarities and differences between domains. This integration is a crucial step to enhance understanding of executive control and behavioral change.

### Beyond action control

Many principles of our action control framework may translate to control in other domains, including emotion and control of thought. Several lines of evidence suggest a certain overlap between control of action and control of thought and emotion. Action control and control of emotion and thought may also be coupled because changes in internal states (such as thoughts or an emotional reaction) could trigger changes in ongoing actions. Corbetta et al.’s (2008) review suggested that the ventral attention network, which is required for action control, might be involved in switching between aspects of “internal” processing, such as memory retrieval or self-referential thoughts. Furthermore, brain areas that are important for action control, such as the right inferior frontal gyrus and the right middle frontal gyrus, may also regulate emotional memories (Depue, Curran, & Banich, 2007) or unwanted thoughts (Benoit & Anderson, 2012; Depue, Burgess, Willcutt, Ruzic, & Banich, 2010). The overlap between action control and emotion regulation is further supported by correlations among rumination, inhibition, and task switching (Whitmer & Banich, 2007). These and other similar findings have led several researchers to propose that similar control mechanisms may be required to regulate various aspects of human behavior (Aron, Robbins, & Poldrack, 2004; Banich et al., 2009; J. R. Cohen & Lieberman, 2010). Although speculative, this overlap could again be partly due to involvement of the abstract rule-like representations in various domains (“do not think,” “do not respond,” and so forth). More generally, we believe that the main difference between domains may be in the content controlled, not in the mechanisms by which control is achieved (see also Logan et al., 2014).

The work of Depue, Banich, and others has suggested that emotion and executive control may influence each other (Depue et al., 2010; Depue et al., 2007; Whitmer & Banich, 2007). This link is further discussed by Pessoa (2009), who proposed a dual-competition framework to account for the effects of low- and high-threat emotional information on executive control. This framework can be integrated with our action control framework. More specifically, on the basis of Pessoa’s framework, we hypothesize that emotional content that is low in threat interferes primarily with attentional selection; by contrast, high-threat information would interfere with action selection as well. We believe that this highlights one of the major benefits of our processing framework: By focusing on the specific mechanisms rather than the general functions, a richer and more detailed picture emerges.

In a similar vein, one could use our framework to examine how motivation, mood, stress, and other state-dependent factors influence action control and flexible behavior. For example, animal studies have shown that the prefrontal cortex is modulated by neurotransmitter systems mediating stress and arousal (Arnsten, 2009; Robbins, 2007). The link between stress and action control is also demonstrated by the finding that people with addictions are prone to failing to suppress drug-seeking behavior in stressful situations (Sinha, 2008). We hypothesize that acute stress could influence action control in at least three different ways: It could lead to a narrowed focus of attention (Chajut & Algom, 2003), it could discourage selection of alternative actions (Goschke, 2000), or it could shift the balance between rule-based and associatively mediated action control (Schwabe, Dickinson, & Wolf, 2011). Given the impact of state-dependent factors on behavioral change, a better understanding of which processes are influenced by factors such as stress could lead to the development of new interventions and improvements in existing ones.

## Conclusion

To conclude, we hope that this article will inspire research on action control, behavioral flexibility, and behavioral change to focus more on specific cognitive processes and representations, how these can be learned throughout development and adulthood, and how these are maintained. We believe that this will lead to better models of executive control of action and behavioral change, which can inspire the development of new and more effective theory-driven interventions. Ultimately, this approach will allow us to banish homunculi from our theories.

## Notes

1. The accumulation process is analogous to drawing a series of balls from a container (Usher & McClelland, 2001). For example, the container can hold balls with “left” or “right” written on them. At each time step in the response-selection process, a ball would be drawn, and a count is added to the appropriate counter (e.g., when a “left” ball is selected, a count is added to the left counter). A response is selected when one of the counters reaches a threshold. When the response threshold is 10, then a response is selected when 10 “left” balls or 10 “right” balls have been drawn from the container.

2. A quasi-experiment was subsequently conducted. The second author tested the prepared reflex idea by either signaling or not signaling at this junction when a car was waiting to turn on another 20 days, signaling on 10 occasions and not signaling on 10 occasions in alternation (this took about half a year). The results were that on 50% of “signal” occasions, the car immediately turned. This never happened on “no-signal” occasions.
